# Buckling and Free Vibration Analyses of Various Nanoparticle Reinforced Concrete Beams Resting on Multi-Parameter Elastic Foundations

**DOI:** 10.3390/ma16175865

**Published:** 2023-08-27

**Authors:** Soumia Dine Elhennani, Zouaoui R. Harrat, Mohammed Chatbi, Asma Belbachir, Baghdad Krour, Ercan Işık, Ehsan Harirchian, Mohammed Bouremana, Mohamed Bachir Bouiadjra

**Affiliations:** 1 Laboratoire des Structures et Matériaux Avancés dans le Génie Civil et Travaux Publics, Djillali Liabes University, Sidi Bel-Abbes 22000, Algeriazouaoui.harrat@dl.univ-sba.dz (Z.R.H.); mohammed.chatbi@dl.univ-sba.dz (M.C.);; 2 Laboratory of Materials and Processes of Construction, Abdelhamid Ibn-Badis University, Mostaganem 27000, Algeria; asma.belbachir@univ-mosta.dz; 3 Department of Civil Engineering, Bitlis Eren University, Bitlis 13100, Turkey; 4 Institute of Structural Mechanics (ISM), Bauhaus-Universität Weimar, 99423 Weimar, Germany; 5 Thematic Agency for Research in Science and Technology (ATRST), Algiers 16000, Algeria

**Keywords:** buckling, vibration, reinforced concrete, beams theory, homogenization model, nanoparticles, Winkler–Pasternak–Kerr foundation

## Abstract

Given their considerable specific surface area and amorphous characteristics, nanoparticles exhibit excellent pozzolanic activity, and when undergoing a reaction with calcium hydroxide, this leads to the generation of a denser matrix by promoting the formation of a greater amount of C-S-H gel, thereby enhancing the strength and durability of the concrete and fortifying the overall structure. Indeed, the present study investigates a comparative study of the buckling and free vibration analyses of concrete beams reinforced with various types of nanoparticles. For its simplicity and accuracy, a higher-order shear deformation theory will be used to analytically model the reinforced concrete beam. Furthermore, the powerful Eshelby’s model is used to derive the equivalent nanocomposite properties. The soil medium is simulated with Pasternak elastic foundation, including a shear layer, and Winkler’s spring, interlinked with a Kerr foundation. The motion equations are derived using Hamilton’s principle. Moreover, based on Navier’s analytical methods, the closed-form solutions of simply supported beams have been obtained. Different parameters, such as type and volume percent of nanoparticles, geometrical parameters, choice of theory and soil medium, on the buckling and dynamic behavior of the beam, are exercised and shown. The major findings of this work indicate that the use of nanoparticles in concretes increases better mechanical resistance and amplifies the natural frequencies. In addition, the elastic foundation has a significant impact on the buckling and vibration performances of concrete beams.

## 1. Introduction

Civil engineering structures are dimensioned for an average lifespan of more than 100 years. Despite this, a number of disorders are to blame for shortening this intended life, and some buildings currently need maintenance to ensure the safety of users. In civil engineering, maintaining structures include safeguarding them by preventing corrosion or guaranteeing proper sealing, repairing them by exploring to make up for resistance losses caused by cracking, and further strengthening them by enhancing the structures’ toughness and performance.

For the design of various technological issues, it is crucial to analyze the free vibration of concrete buildings. Engineers need to be aware of the frequencies and related modes in order to properly design the system due to the vibratory effect on equipment that is performance-sensitive and mounted on concrete beams. Additionally, as concrete is the most practical material for building structures, its quality must be improved to lessen vibrations. By enhancing the characteristics of concrete beams with the inclusion of nanomaterials to raise their rigidities, it is now possible to reduce free vibrations thanks to the advancements that nanotechnologies may offer to building materials.

Over the past two decades, there has been a significant increase in the produced work in the field of reinforced concretes with nanoparticles. Some examples of the experimental investigations on the effect of nanoparticles on concretes are cited herein. Mirzadeh [1] employed an artificial neural network (ANN) to predict the impacts of nano-silica, water/binder ratio, sand/binder ratio, and aging (curing) duration on the compressive strength of cement mortars. Their established model serves as a practical tool for preliminary estimation during the mix design phase, facilitating the production of cost-effective and superior-quality cement mortars. Rupasinghe et al. [2] formulated a multiscale finite element model by quantifying microstructural image analysis, enabling the prediction of compressive strength for the nano-modified system. They employed a numerical approach to assess the compressive strength of cement systems integrated with nano-silica. Oltulu and Şahin [3] explored the impact of individual and combined nano-SiO_2_, nano-Al_2_O_3_, and nano-Fe_2_O_3_ powders on the compressive strengths and capillary water absorption of cement mortar incorporating fly ash. The most favorable outcomes were achieved in the group of mortars supplemented with a combination of NS, NA, and NF powders at a concentration of 1.25 wt.%. Al-Jadiri et al. [4] studied the effect of zirconium oxide nanoparticles on the microstructural development of cement mortars. According to the SEM analysis, mortars with ZrO_2_ revealed a microstructure with a high compaction degree and an increase in compression strength of 9%. Li et al. [5] explored the impact of titanium dioxide nanoparticles on concrete. The findings indicated that when the weight ratio of TiO_2_ nanoparticles in concrete reaches 2 wt.%, the compressive strength of the concrete reaches its maximum, exhibiting a 7% increase compared to concrete without TiO_2_ nanoparticle additives.

Mathematically speaking, there are very few investigations on the impact of nanoparticles on reinforced concrete. Zamanian et al. [6] studied agglomeration impact on the buckling response of embedded concrete columns reinforced with SiO_2_ nano-particles. Numerical results indicate that considering the agglomeration effect leads to the decrease in buckling load of structure. Shokravi [7] used Timoshenko’s first-order shear theory, Mori–Tanaka’s homogenization model, and DQM method to predict the frequencies of concrete beams reinforced with SiO_2_ nanoparticles. The same author [8] studied buckling control of concrete beams armed by zinc oxide (ZnO) nanoparticles, and showed that increasing the ZnO nanoparticles leads to improving the buckling load. Alijani et al. [9] used a higher-order shear deformation theory to simulate a concrete foundation reinforced by SiO_2_ nanoparticles resting on soil bed, and by the means of analytical solutions, frequency of the system has been calculated. Harrat et al. [10] studied the static behavior of nano-SiO_2_ reinforced concrete beams using a simple two-phase homogenization model, when Chatbi et al. [11] also conducted an analytical investigation on the buckling behavior of nano-SiO_2_ enhanced concrete plates resting on Kerr’s 3-parameter elastic foundation. The predominant outcomes of those studies suggest that the incorporation of an optimal quantity of SiO_2_ nanoparticles enhances the mechanical properties of concrete. Furthermore, they deduced that the elastic foundation significantly influences the bending behavior of concrete structures. The collective findings of the research consistently indicate that the addition of SiO_2_ nanoparticles contributes to increased mechanical strength of concrete, leading to reduced strains and deflections. In a related study, the same researchers undertook an analytical investigation into the mechanical behavior of concrete beams reinforced with various clay nanoparticles (NCs) [12]. Additionally, they analyzed the thermomechanical bending of concrete beams reinforced with ferric oxide nanoparticles [13]. These inquiries notably emphasized the ability of these additives to act as enhancers for concrete beams when subjected to external bending loads.

To overcome the limitations of classical theories, many researches are seeking ways to develop new higher-order shear deformation theories. Atmane et al. [14] employed a computational shear displacement model for the vibration analysis of functionally graded beams with porosities. This model operates under the assumption that the transverse displacements can be separated into bending and shear components. Notably, the bending components do not contribute to shear forces, and similarly, the shear components do not contribute to bending moments. Ebrahimi et al. [15] investigated the thermo-mechanical vibration analysis of functionally graded micro-/nano-scale beams with porosities employing a refined hyperbolic beam theory that validates the shear deformation effect at the top and bottom surfaces of the beam, eliminating the need for a shear correction factor. Bensaid et al. [16] examined the free vibration properties of nano-scale beams supported by an elastic Pasternak’s foundation. They employed a nonlocal strain gradient theory and a higher-order hyperbolic beam model to account for shear deformation effects without the need for a shear correction factor.

To the best of the authors’ knowledge, the literature lacks comprehensive analytical comparative studies assessing the performance of concrete beams reinforced with a variety of nanoparticles. Addressing this gap, our aim is to introduce a mathematical model for simulating concrete beams enriched with either silicon dioxide, titanium dioxide, or zircon monoxide nanoparticles. Our primary objective is to enhance both the dynamic characteristics and mechanical properties of concrete structures by strategically integrating these entities within the concrete matrix. Additionally, our investigation thoroughly considers the distinct properties of these nanoparticles, with the aim of revealing their unique potential as enhancers for concrete. This analysis also seeks to accurately identify the nanoparticles that exhibit the highest efficacy in improving both the dynamic and static behaviors of concrete beams. This comprehensive exploration advances our understanding and highlights the pivotal role that nanoparticles play in elevating the overall structural performance of concrete.

To ensure robustness and reliability, our structural modeling employs a higher-order shear deformation theory, forming the basis for deriving governing equations through Hamilton’s principle. Utilizing Navier’s analytical technique, we meticulously dissect the impact of multiple parameters—nanoparticle type, volume percentage, geometric dimensions, and the influence of an elastic foundation—on the buckling and dynamic analyses of the beam. This comprehensive exploration stands as a testament to our dedication to advancing the understanding and application of nanoparticle reinforced concrete, ushering in a new era of innovation in construction materials.

## 2. Technology of Nanoparticles

This chapter offers a brief overview with an emphasis on the application of nanomaterials in alkali-activated materials. Due to the improved capabilities of nanoparticles, it has been shown that the use of nanomaterials in alkali-activated materials has tremendous promise for the construction industry. Due to its nucleation effect to accelerate the cement hydration process and pozzolanic activity [17], nano-silica (NS) has been widely used in Portland cement concrete to improve microstructure [18], mechanical properties [19], and durability [20,21]. Another justification for using nano-silica in place of cement in concrete is to lessen its carbon footprint [22]. Due to the numerous increased qualities brought about by nano-silica, it has also recently attracted an increasing amount of interest to utilize it in alkali-activated systems.

**Figure 1:**
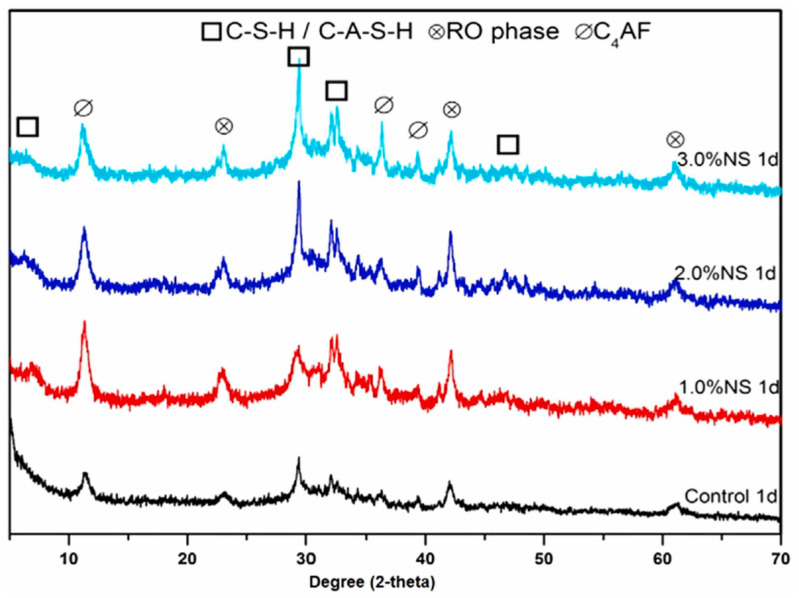
XRD of samples cured for 1 day with different dosages of SiO_2_ [18].

The XRD patterns shown in Figure 1 of the geopolymer pastes incorporating nano-SiO_2_ are comparable to that of fly ash. Notably, an increase in quartz content is observed, which can be attributed to the introduction of nano-silica. The presence of the C-S-H phase is confirmed by the presence of peaks at 29.5° and 32.05° 2-theta, highlighting its contribution to the mechanical characteristics of the geopolymer matrix.

**Figure 2:**
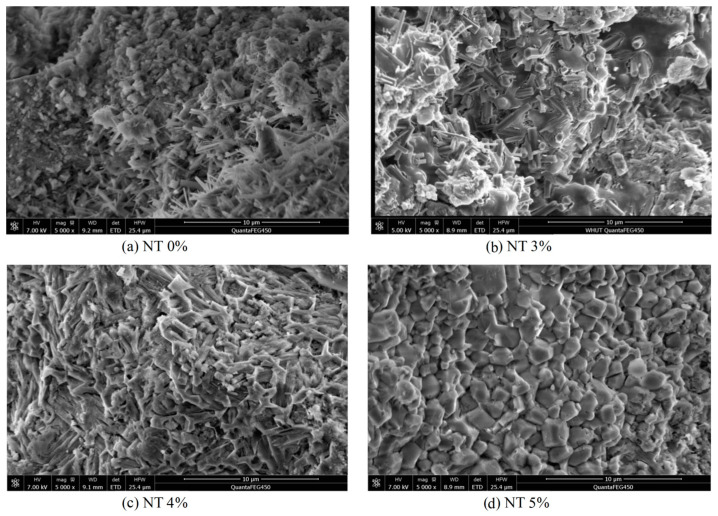
SEM images of mortars with nano-titanium [27].

Nano-TiO_2_ has been extensively researched due to its many useful qualities, including its ability to filter the air and act as a self-cleaning agent [23,24,25,26]. Additionally, the inclusion of nano-TiO_2_ to concretes refines the microstructure, as evidenced by SEM images in Figure 2. This refinement contributes to improved mechanical properties and a reduction in interior micro-cracks [27].

Yang et al. [28] investigated how alkali-activated slag’s strength, shrinkage, and microstructure were impacted by nano-TiO_2_. As the alkali activator, a mixture of solid NaOH and liquid water glass with a 9.7% Na_2_O concentration was utilized. A mass ratio of 0.5% of nano-TiO_2_ was introduced to the system, with particle sizes ranging from 20 to 100 nm. According to the FTIR data [28], the additional nano-TiO_2_ enhances transmittance at 1420, 946, and 457, which suggests that more hydration products (C-S-H and C-A-S-H gels) were formed. This is consistent with the denser microstructure that SEM examination revealed in samples that included TiO_2_.

## 3. Homogenization (Eshelby’s Model)

**Figure 3:**
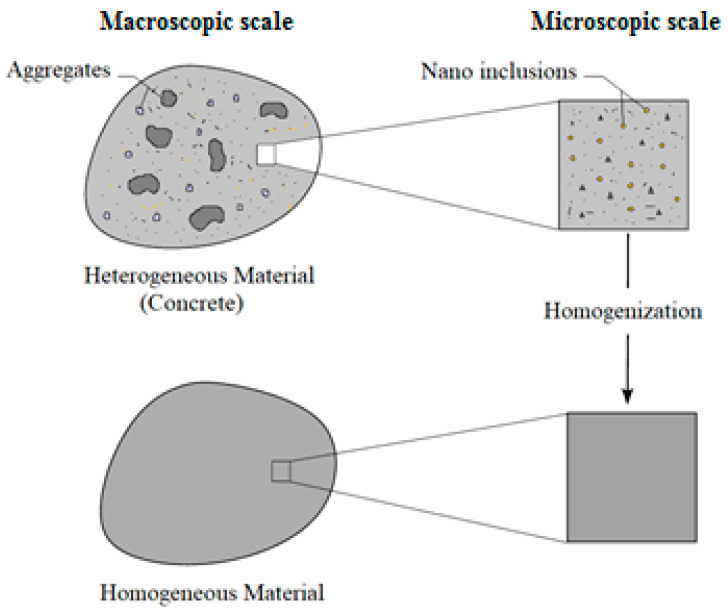
Homogenizing nanometric inclusions in a concrete matrix.

When a matrix is infused with nanoparticles of varying physical and mechanical properties, resulting in a biphasic composite commonly referred to as a nanocomposite, the material inherently possesses heterogeneous characteristics. To homogenize these properties effectively, a robust and reliable model is required. Eshelby’s homogenization model is widely recognized and recommended for these biphasic nanocomposites [29]. In this context, the nano-sized inclusions occupy a designated subvolume denoted as *V_r_*. In our analysis, these inclusions are assumed to have spherical shapes. The matrix (concrete), encompassing the complementary volume *V_m_*, is assumed to extend infinitely, ensuring that remote boundaries remain free of traction and displacement. The inclusions (varieties of nanoparticles, in our case) undergo an inelastic strain known as eigenstrain. This eigenstrain emulates the strain experienced by the nanoparticle when it contracts under load-free conditions, unaffected by any matrix constraints. This interplay creates self-equilibrated residual stress fields within both the inclusion and the matrix, as depicted in Figure 3. This section presents the general expression for the Eshelby tensor, a versatile mathematical tool used to describe inclusions of diverse topological shapes. Moreover, the Eshelby tensor for a spherical/ellipsoidal inclusion can be calculated through numerical methods.

Nano-SiO_2_, -TiO_2_, -ZrO are chosen for their spherical shape of inclusion in order that the Eshelby model could be used to calculate the buckling load and the frequencies of nanoparticle reinforced concrete beams.

Eshelby’s homogenization model stands out as the predominant analytical framework for predicting the properties of a matrix reinforced with nanocomposites. Notably, this model is applicable under the condition of a spherical/ellipsoidal inclusion embedded within an infinite matrix [30], the nanoparticles considered in this study are treated as spherical in physical shape. The stiffness tensor *C^T^* of the nanocomposite is given in Equation (1):

(1) $$C^{T}={\left(C_{m}^{-1}-V_{r}{\left\{\left(C_{r}-C_{m}\right)\left[S-V_{r}\left(S-I\right)\right]+C_{m}\right\}}^{-1}\left(C_{r}-C_{m}\right)C_{m}^{-1}\right)}^{-1}$$

In Equation (1), *I* represents the identity matrix, *C_m_* and *C_r_* correspond to the stiffness tensors of the concrete matrix and the nano-inclusion reinforcement, respectively. While, *V_m_* and *V_r_* denote the volume fraction of the matrix and nano-sized reinforcement, while *S* represents the Eshelby tensor, which is influenced by the Poisson’s ratio of reinforcements.

Once again, for both isotropic materials (concrete matrix and nano-sized reinforcements) and according the well-known conversion formula, the stiffnesses *C_m_* and *C_r_* are formulated in Equation (2):

(2a) $$C_{11}=C_{22}=\frac{\left(1-\upsilon \right)E}{\left(1+\upsilon \right)\left(1-2\upsilon \right)}$$

(2b) $$C_{12}=\frac{\upsilon E}{\left(1+\upsilon \right)\left(1-2\upsilon \right)}$$

(2c) $$C_{44}=C_{55}=C_{66}=\frac{E}{\left(1+\upsilon \right)}$$

In which *E* is the elastic Young’s modulus of either the concrete matrix or the nano-sized particles incorporations, and *υ* denotes the Poisson’s ratio. The subscripts 1, 2, 3 correspond to x, y, z directions of the composite Cartesian coordinate system, respectively.

For the incorporated reinforcements that are supposed to be spherically shaped, Eshelby’s tensor *S* is presented in Equation (3) as [31]:

(3) $$S=\left[\begin{array}{cccccc} S_{1111} & S_{1122} & S_{1133} & S_{1123} & S_{1113} & S_{1112}\\ S_{2211} & S_{2222} & S_{2233} & S_{2223} & S_{2213} & S_{2212}\\ S_{3311} & S_{3322} & S_{3333} & S_{3323} & S_{3313} & S_{3312}\\ S_{2311} & S_{2322} & S_{2333} & S_{2323} & S_{2313} & S_{2312}\\ S_{1311} & S_{1322} & S_{1333} & S_{1323} & S_{1313} & S_{1312}\\ S_{1211} & S_{1222} & S_{1233} & S_{1123} & S_{1213} & S_{1212} \end{array}\right]$$

where:

(4a) $$\begin{array}{l} S_{1111}=S_{2222}=0;\;S_{3333}=1\\ S_{1122}=S_{1133}=S_{2233}=S_{2211}=0;\;S_{3311}=S_{3322}=\frac{\upsilon_{r}}{\left(1-\upsilon_{r}\right)}\\ S_{1212}=0;\;S_{1313}=S_{2323}=1/2 \end{array}$$

For all other components:

(4b) $$S_{ijkl}=0$$

Here, *υ_r_* represents the Poisson’s ratio of nano-sized particles reinforcements.

## 4. Mathematical Modeling

It is noted that the results evaluated using the classical beam theory of short beams may not be reliable [32]. As a result, this work uses a higher-order shear deformation theory ‘HSDT’ to analyze the buckling and free vibration behaviors of beams made of concrete reinforced with nanoparticles.

**Figure 4:**
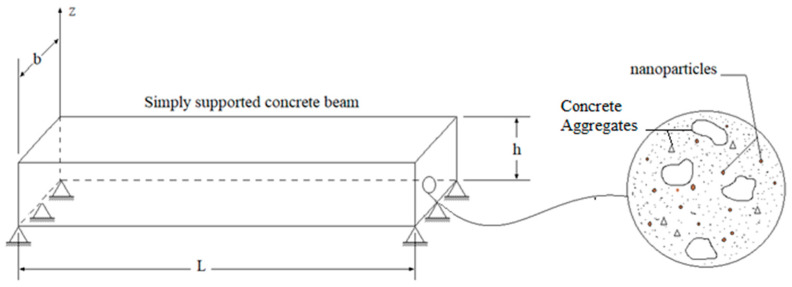
Geometric configuration of a nanoparticle reinforced simply supported concrete beam.

The reinforced concrete beam is assumed to possess dimensions of length ‘*L*’, width ‘*b*’, and total thickness ‘*h*’, as shown in Figure 4. It is also considered to be fully simply supported at the edges, *x* = 0 and *x* = *L*.

The embedded nanoparticles are distributed randomly within the concrete matrix, as depicted in Figure 4. The coordinate system noted (*x*, *y*, *z*) on which *z* is placed in the median plane of the beam:

0≤x<L; 0≤y<b; −h/2≤z<h/2

### 4.1. Kinematics

The HSDT displacement field of a material point situated at coordinates (*x*, *y*, and *z*) within the beam can be described as follows:

(5a) $$u(x,y,z)=u_{0}(x,y)-z\frac{\partial w_{b}(x,y)}{\partial x}-f(z)\frac{\partial w_{s}(x,y)}{\partial x}$$

(5b) $$w(x,y,z)=w_{b}(x,y)+w_{s}(x,y)$$

Here, the axial displacement is denoted as ‘*u*’, and the transverse displacement at the reference plane of the system is represented by ‘*w*’, where ‘*f(z)*’ is a shape function utilized to characterize the distribution of transverse shear stress across the thickness of the structure. The specific shape functions for various higher-order shear theories are provided in Table 1.

Throughout our analytical investigation, we systematically examined a selection of higher-order shear deformation theories outlined in Table 1 to simulate the behavior of the nano-reinforced concrete beam. The deliberate inclusion of these different deformation theories was encouraged by the aim of enhancing the precision and accuracy of our analytical modeling, thereby refining our overall approach. By incorporating this diverse array of deformation theories, we were able to thoroughly assess and compare their individual performances, ensuring the reliability and robustness of our analysis.

The linear strain components can be derived from the displacement field in Equation (5) as:

(6a) $$\varepsilon_{x}=\frac{\partial u}{\partial x}=\varepsilon_{x}^{0}+zk_{x}^{b}+f\left(z\right)k_{x}^{s}$$

(6b) $$\gamma_{xz}=\frac{\partial u}{\partial x}+\frac{\partial w}{\partial x}=g(z)\gamma_{xz}^{s}$$

where:

(7) $$\varepsilon_{x}^{0}=\frac{\partial u_{0}}{\partial x};\;k_{x}^{b}=-\frac{\partial^{2}w_{b}}{\partial x^{2}};\;k_{x}^{s}=-\frac{\partial^{2}w_{s}}{\partial x^{2}};\;\gamma_{xz}^{s}=\frac{\partial w_{s}}{\partial x};\;g(z)=1-\frac{\partial f(z)}{\partial z}.$$

The constitutive stress–strain relations of the nanocomposite can be defined as:

(8) $$\left\{\begin{array}{c} \sigma_{x}\\ \tau_{xz} \end{array}\right\}=\left[\begin{array}{cc} Q_{11} & 0\\ 0 & Q_{55} \end{array}\right]\left\{\begin{array}{c} \varepsilon_{x}\\ \gamma_{xz} \end{array}\right\}$$

where *Qij* are the elastic constants which can be obtained using Eshelby’s model.

### 4.2. Equations of Motion

The virtual work’s principle is applied to develop the equations of motion:

(9) $$\int_{t1}^{t2}\left({\delta U}_{b}+{\delta W}_{k}+\delta \Phi -\delta K\right)\partial t=0$$

where *δU_b_* and *δW_k_* are the virtual variation of the internal strain energy of the beam and foundation, respectively, *δΦ* is the virtual work carried out by external forces, while *δK* is the virtual kinetic energy.

Firstly, the expression of the virtual strain energy carried out by the beam is:

(10) $${\delta U}_{b}=\int_{0}^{L}\int_{A}^{}\left(\sigma_{x}\delta \varepsilon_{x}+\tau_{xz}\delta \gamma_{xz}\right)dAdx$$

Submitting Equation (6) into Equation (10), the potential energy is given as follows:

(11) $${\delta U}_{b}=\int_{0}^{L}\left(N\frac{\partial \delta u_{0}}{\partial x}-M_{b}\frac{\partial^{2}\delta w_{b}}{\partial^{2}x}-M_{s}\frac{\partial^{2}\delta w_{s}}{\partial^{2}x}+Q\frac{\partial \delta w_{s}}{\partial x}\right)dx$$

where:

(12a) $$N=\int_{-h/2}^{h/2}\sigma_{x}bdz$$

(12b) $$M_{b}=\int_{-h/2}^{h/2}{z\sigma }_{x}bdz$$

(12c) $$M_{s}=\int_{-h/2}^{h/2}f(z)\sigma_{x}bdz$$

(12d) $$Q=\int_{-h/2}^{h/2}g(z)\tau_{xz}bdz$$

‘*N*’ represents the normal force in a structural element, ‘*M_b_*’ denotes the bending moment of the beam’s local y-axis, ‘*M_s_*’ indicates the shear force acting along the beam’s local z-axis, and ‘*Q*’ represents the torsional moment applied to the beam.

By substituting Equation (6) into Equation (8), and the results into Equation (12), one obtains the stress resultants in the form of material stiffness and displacement components:

(13a) $$N=A\frac{\partial u_{0}}{\partial x}-B\frac{\partial^{2}w_{b}}{\partial x^{2}}-B_{s}\frac{\partial^{2}w_{s}}{\partial x^{2}}$$

(13b) $$M_{b}=B\frac{\partial u_{0}}{\partial x}-D\frac{\partial^{2}w_{b}}{\partial x^{2}}-D_{s}\frac{\partial^{2}w_{s}}{\partial x^{2}}$$

(13c) $$M_{s}=B_{s}\frac{\partial u_{0}}{\partial x}-D_{s}\frac{\partial^{2}w_{b}}{\partial x^{2}}-H_{s}\frac{\partial^{2}w_{s}}{\partial x^{2}}$$

(13d) $$Q=A_{s}\frac{\partial w_{s}}{\partial x}$$

where ($A,B,D,B_{s},D_{s},H_{s})$ are the beam stiffness, defined by:

(14a) $$\left\{A,B,D,B_{s},D_{s},H_{s}\right\}=\int_{-h/2}^{h/2}\left\{1,z,z^{2},f\left(z\right),zf\left(z\right),f(z){}^{2}\right\}Q_{11}dz$$

(14b) $$\left\{A_{s}\right\}=\int_{-h/2}^{h/2}\left\{g(z){}^{2}\right\}Q_{11}dz$$

For the RC beam under axial in-plane compressive buckling load ‘*N*_0*x*_’, the virtual work carried out by the external loading is:

(15) $$\delta \phi =-\int_{A}^{}\delta \left(N_{0x}\left(\frac{\partial {}^{2}\left(w_{b}+w_{s}\right)}{\partial x{}^{2}}\right)\right)dA$$

The expression of the virtual strain energy carried out by the foundation is:

(16) $$\delta W_{k}=-\int_{A}^{}QdA$$

Kerr’s foundation is a 3-parameter model. An upper layer $K_{U}$, a shear layer $K_{S}$, and a lower layer $K_{L}$ are considered and can be expressed as:

(17) $$Q_{kerr}=\frac{K_{L}K_{U}}{K_{L}+K_{U}}\left(w_{b}+w_{s}\right)-\frac{K_{S}K_{U}}{K_{L}+K_{U}}\left(\frac{\partial {}^{2}w_{b}}{\partial x{}^{2}}+\frac{{\partial^{2}w}_{s}}{\partial x{}^{2}}\right)$$

The Kerr foundation becomes Pasternak foundation by taking infinite upper layer $K_{U}$ stiffness, in which Pasternak foundation can be expressed as:

(18) $$Q_{Pasternak}=K_{L}\left(w_{b}+w_{s}\right)-K_{S}\left(\frac{\partial {}^{2}w_{b}}{\partial x{}^{2}}+\frac{{\partial^{2}w}_{s}}{\partial x{}^{2}}\right)$$

whereas without elastic foundation: $K_{S}=K_{L}=K_{U}=0$.

The variation of the kinetic energy of the beam is calculated by:

(19) $$\delta K=\int_{0}^{L}\int_{A}^{}\left[\dot{u_{0}}\delta \dot{u_{0}}+\dot{w_{b}}\delta \dot{w_{b}}+\dot{w_{s}}\delta \dot{w_{s}}\right]\rho \left(z\right)dAdx$$

(20) $$\delta K=\int_{0}^{L}\left\{I_{0}\left[\dot{u_{0}}\delta \dot{u_{0}}+\left((\dot{w_{b}}+\dot{w_{s}}\right)(\delta \dot{w_{b}}+\delta \dot{w_{s}})\right]-I_{1}\left(\dot{u_{0}}\frac{\partial \delta \dot{w_{b}}}{\partial x}+\delta \dot{u_{0}}\frac{\partial \delta \dot{w_{b}}}{\partial x}\right)+I_{2}\frac{\partial \dot{w_{b}}}{\partial x}\frac{\partial \delta \dot{w_{b}}}{\partial x}\;-J_{1}\left(\dot{u_{0}}\frac{\partial \delta \dot{w_{s}}}{\partial x}+\delta \dot{u_{0}}\frac{\partial \dot{w_{s}}}{\partial x}\right)+K_{2}\frac{\partial \dot{w_{s}}}{\partial x}\frac{\partial \delta \dot{w_{s}}}{\partial x}+J_{2}\left(\frac{\partial \dot{w_{b}}}{\partial x}\frac{\partial \delta \dot{w_{s}}}{\partial x}+\frac{\partial \dot{w_{s}}}{\partial x}\frac{\partial \delta \dot{w_{b}}}{\partial x}\right)\right\}$$

where:

(21) $$\left\{I_{0},I_{1},J_{1},I_{2},J_{2},K_{2}\right\}=\int_{-h/2}^{h/2}\left\{1,z,f(z),z{}^{2},zf\left(z\right),f(z){}^{2}\right\}\rho dz$$

By substituting Equations (11), (15), and (16) into Equation (9), then, integrating by parts and collecting the coefficients of *δu_0_, δw_b_,* and *δw_s_*, engender the following equations of motion:

(22a) $$\delta u_{0}:\;\frac{\partial N}{\partial x}=I_{0}u_{0}-I_{1}\frac{{\partial w}_{b}}{\partial x}-J_{1}\frac{\partial w_{s}}{\partial x}$$

(22b) $$\delta w_{b}:\;\frac{\partial^{2}M_{b}^{}}{\partial x^{2}}+Q+N_{0x}\left(\frac{{\partial^{2}w}_{b}}{\partial x{}^{2}}+\frac{{\partial^{2}w}_{s}}{\partial x{}^{2}}\right)=I_{0}\left(w_{b}+w_{s}\right)+I_{1}\frac{\partial u_{0}}{\partial x}-I_{2}\frac{\partial^{2}w_{b}}{\partial x^{2}}-J_{2}\frac{\partial^{2}w_{s}}{\partial x^{2}}$$

(22c) $$\delta w_{s}:\;\frac{\partial^{2}M_{s}^{}}{\partial x^{2}}+\frac{\partial Q}{\partial x}-Q+N_{0x}\left(\frac{{\partial^{2}w}_{b}}{\partial x{}^{2}}+\frac{{\partial^{2}w}_{s}}{\partial x{}^{2}}\right)=I_{0}\left(w_{b}+w_{s}\right)+J_{1}\frac{\partial u_{0}}{\partial x}-J_{2}\frac{\partial^{2}w_{b}}{\partial x^{2}}-K_{2}\frac{\partial^{2}w_{s}}{\partial x^{2}}$$

where:

(23a) $$A\frac{\partial^{2}u_{0}}{\partial x^{2}}-B\frac{\partial^{3}w_{b}}{\partial x^{3}}-B_{s}\frac{\partial^{3}w_{s}}{\partial x^{3}}=I_{0}u_{0}-I_{1}\frac{\partial w_{b}}{\partial x}-J_{1}\frac{\partial w_{s}}{\partial x}$$

(23b) $$B\frac{\partial^{3}u_{0}}{\partial x^{3}}-D\frac{\partial^{4}w_{b}}{\partial x^{4}}-D_{s}\frac{\partial^{4}w_{s}}{\partial x^{4}}+Q+N_{0x}\left(\frac{{\partial^{2}w}_{b}}{\partial x{}^{2}}+\frac{{\partial^{2}w}_{s}}{\partial x{}^{2}}\right)=I_{0}\left(w_{b}+w_{s}\right)+I_{1}\frac{\partial u_{0}}{\partial x}-I_{2}\frac{\partial^{2}w_{b}}{\partial x^{2}}-J_{2}\frac{\partial^{2}w_{s}}{\partial x^{2}}$$

(23c) $$B_{s}\frac{\partial^{3}u_{0}}{\partial x^{3}}-D_{s}\frac{\partial^{4}w_{b}}{\partial x^{4}}-H_{s}\frac{\partial^{4}w_{s}}{\partial x^{4}}+A_{s}\frac{\partial^{2}w_{s}}{\partial x^{2}}-Q+N_{0x}\left(\frac{{\partial^{2}w}_{b}}{\partial x^{2}}+\frac{{\partial^{2}w}_{s}}{\partial x^{2}}\right)=I_{0}\left(w_{b}+w_{s}\right)+J_{1}\frac{\partial u_{0}}{\partial x}-J_{2}\frac{\partial^{2}w_{b}}{\partial x^{2}}-K_{2}\frac{\partial^{2}w_{s}}{\partial x^{2}}$$

### 4.3. Closed-Form Solutions for Simply Supported Beams

Navier’s admissible displacement functions in the form of trigonometric series which satisfy the boundary condition of the problems are given below:

(24a) $$u_{0}(x,t)=\sum_{n=1}^{\infty }U_{n}e^{i\omega t}cos\left(\lambda x\right)$$

(24b) $$w_{b}(x,t)=\sum_{n=1}^{\infty }W_{bn}e^{i\omega t}sin\left(\lambda x\right)$$

(24c) $$w_{s}(x,t)=\sum_{n=1}^{\infty }W_{sn}e^{i\omega t}sin\left(\lambda x\right)$$

Finally, in order to obtain the analytical solutions, the results of the substitution can be arranged into the following matrix form:

(25) $$\left(\left[\begin{array}{ccc} S_{11} & S_{12} & S_{13}\\ S_{21} & S_{22} & S_{23}\\ S_{31} & S_{32} & S_{33} \end{array}\right]-\omega^{2}\left[\begin{array}{ccc} m_{11} & m_{12} & m_{13}\\ m_{21} & m_{22} & m_{23}\\ m_{31} & m_{32} & m_{33} \end{array}\right]\right)\left\{\begin{array}{c} U_{n}\\ W_{bn}\\ W_{sn} \end{array}\right\}=\left\{\begin{array}{c} 0\\ 0\\ 0 \end{array}\right\}$$

where the rigidities can be expressed as:

(26a) $$S_{11}=-A\lambda^{2};\;S_{12}=B{\lambda^{3};\;S}_{13}={B_{s}\lambda }^{3}$$

(26b) $$S_{21}=S_{12}{;\;S}_{22}={D\lambda }^{4}+\frac{K_{L}K_{U}}{K_{L}+K_{U}}+\frac{K_{S}K_{U}}{K_{L}+K_{U}}\lambda^{2};\;S_{23}={D_{s}\lambda }^{4}+\frac{K_{L}K_{U}}{K_{L}+K_{U}}+\frac{K_{S}K_{U}}{K_{L}+K_{U}}\lambda^{2}$$

(26c) $$S_{31}=S_{13};\;S_{32}=S_{23}{;\;S}_{33}={{-H}_{s}\lambda }^{4}-A_{s}\lambda^{2}-\frac{K_{L}K_{U}}{K_{L}+K_{U}}-\frac{K_{S}K_{U}}{K_{L}+K_{U}}\lambda^{2}$$

and the mass constants can be given as follows:

(27a) $$m_{11}=I_{0};\;m_{12}={-I}_{1}\lambda {;\;m}_{13}={-J}_{1}\lambda$$

(27b) $$m_{21}=m_{12}{;\;m}_{22}=I_{0}{+I}_{2}\lambda^{2}{;\;m}_{23}=I_{0}+J_{2}\lambda^{2}$$

(27c) $$m_{31}=m_{13};\;m_{32}=m_{23}{;\;m}_{33}=I_{0}{+K}_{2}\lambda^{2}$$

## 5. Results and Discussion

In the following sections, a range of analytical simulations are presented and discussed regarding the static (buckling analysis) and dynamic (free vibration analysis) responses of concrete beams reinforced with various types of amorphous nanoparticles. For this purpose, a computer program is coded to simulate the beam structure considering the assumptions of the refined beam theory (RBT). It should be noted that the relevance of this work lies in the use of different types of nanoscale reinforcements for a concrete matrix to compare the effect of each in terms of mechanical and physical effectiveness.

For this, a concrete matrix with a modulus of elasticity of $E_{m}=20\;GPa$, a density of $\rho_{m}=2400\;Kg/m^{3}$, and a Poisson’s ratio $\nu_{m}=0.24$, are used for the analytical computations.

The reinforcement nanoparticles incorporated in the beam were varied for the same concrete matrix. We selected three nano-sized particles with different nature and characteristics as shown in Table 2.

The following dimensionless parameters are used in presenting the numerical results in graphical forms.

- For vibration analysis:

(28) $$\hat{\omega }=\frac{\omega \cdot L^{2}}{h}\sqrt{\frac{\rho_{m}}{\rho_{m}}}$$

- For buckling analysis:

(29) $$N_{cr}=\frac{N_{0x}\cdot L^{2}}{E_{m}\cdot h^{2}}$$

### 5.1. Validation

It is important to first verify the accuracy of the present mathematical model, since numerical results for buckling and dynamic analyses of concrete structures reinforced with TiO_2_ and ZrO nanoparticles are not available in the literature. Considering the material and the geometric parameters similar to Harrat et al. [10], the results in terms of transverse displacements ($\bar{w}$) as well as normal and shear stress ($\bar{\sigma_{x}},\bar{\tau_{xz}}$) of nano-SiO_2_ reinforced concrete beams are adopted to compare with the present results as shown in Table 3.

The comparison of the results presented elucidate that the different beam theories are in absolute agreement with the predicted transverse displacement ($\bar{w}$), and normal end shear stresses ($\bar{\sigma_{x}},\bar{\tau_{xz}}$). Nevertheless, the higher-order shear deformation theories predict accurate results than the classical beam theory.

**Figure 5:**
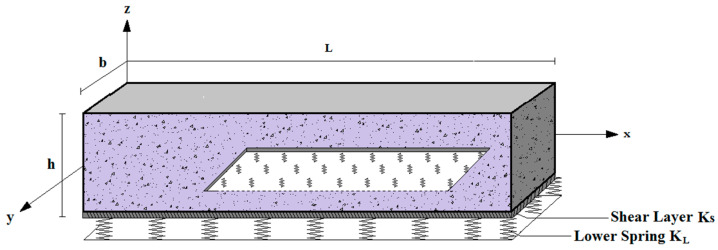
Geometry of a beam resting on a 2-parameter foundation (Winkler–Pasternak foundation).

Figure 5 embodies a nanoparticle reinforced concrete beam on an elastic Winkler–Pasternak foundation. This type of elastic foundation contains a shear layer of stiffness constant *K_S_*, and bound Winkler springs of stiffness *K_L_*.

**Figure 6:**
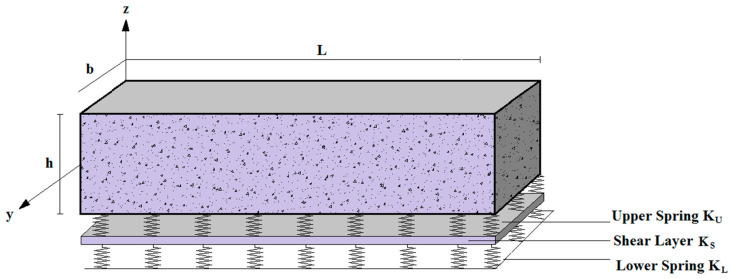
Geometry of a beam resting on a 3-parameter foundation (Kerr foundation).

To broaden the scope of this study, we will further assume that the reinforced concrete beam rests on Kerr’s 3-parameter elastic foundation (Figure 6) by introducing the elastic parameters of the upper springs that Kerr added to the Winkler–Pasternak foundation. It is worth pointing out that in order to reduce the computation rate, the lower spring stiffness (*K_L_*) is set to a constant value (*K_L_* = 10).

### 5.2. Buckling Analysis

In this section, we initiate an analytical analysis utilizing the refined beam theory (RPT) to explore buckling instabilities within a concrete beam enhanced by a range of nano-sized particles. The beam is assumed to be subjected to in-plane axial compressive loading parallel to the lengthwise direction of the beam ‘x’.

In Table 4, the critical buckling loads ($N_{cr}$) of a simply supported concrete beam enhanced with titanium dioxide (TiO_2_) and zirconium oxide (ZrO) is determined by varying the reinforcement volume (*V_r_*) from 0% (non-reinforced beam) to 30% reinforcement out of the total weight of the nanocomposite. Since there are no numerical results in the literature regarding the buckling analysis of TiO_2_ and ZrO nanoparticulated concrete beams, several beam theories, namely, Reddy’s [33] third-order shear deformation theory (TSDT), Touratier [34] trigonometric shear deformation theory, and Karama et al. [35] exponential shear deformation theories, were employed for the accuracy assurance of the results.

Comparing the results given in Table 4, one finds that the different higher-order deformation theories (TSDT, TRSDT, ESDT), predict almost the same critical buckling loads, while the first-order deformation theory (FSDT) and the classical beam theory (CPT), slightly underestimate them. More importantly, it can be clearly observed that the critical buckling load ($N_{cr}$) required to make the beam statically unstable is larger when the concrete beam is reinforced with 30% titanium dioxide than the non-reinforced beam. However, for the subsequent phases of our study, we proceeded with the trigonometric shear deformation theory to ensure the reliability and rigor of our analysis.

As far as Table 4 is concerned, it shows the effect of several volume concentrations (*Vr*) in a simply supported concrete beam (*L*/*h* = 10) subjected to uni-axial buckling loads. It can be deduced from the results that the critical buckling load increases as the reinforcement volume in the concrete matrix increases, making the beam more resilient to the buckle.

**Figure 7:**
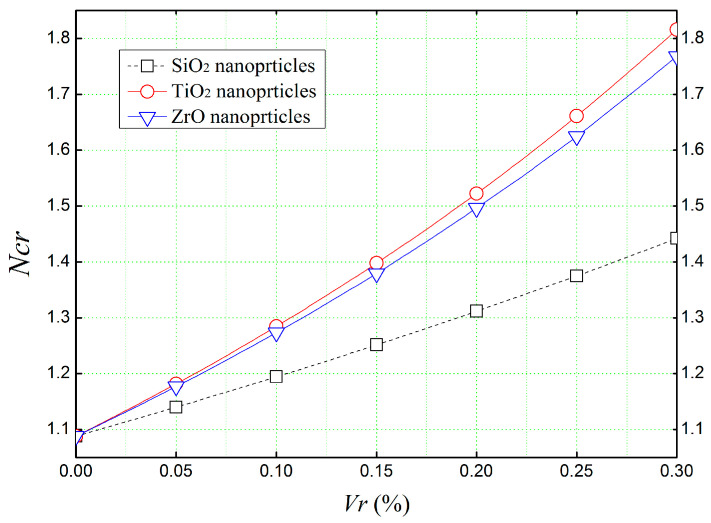
The non-dimensional critical buckling load (*N_cr_*) of simply supported concrete beams reinforced with several types of nanoparticles (*L*/*h* = 4).

To analyze the mechanical influence of different nanoparticles incorporated in a concrete matrix, and in order to determine the most appropriate reinforcement to increase the mechanical resistance against buckling loads, the effect of different volume fractions (*V_r_*) of silica dioxide (SiO_2_), titanium dioxide (TiO_2_), and zirconium oxide (ZrO) on the critical buckling load ($N_{cr}$) of a simply supported concrete beam was illustrated in Figure 7.

It can be apparent from Figure 7 that, all kinds of reinforcements (SiO_2_, TiO_2_, ZrO) have a strengthening effect on the beam, as the critical buckling load ($N_{cr}$) required to make the beam unstable increases with the volume of reinforcement in the concrete matrix. Furthermore, the use of titanium nanoparticles (TiO_2_) in the concrete matrix seems to give even more strength to the beam and makes it more resistant to in-plane buckling loads compared to other types of reinforcements.

**Figure 8:**
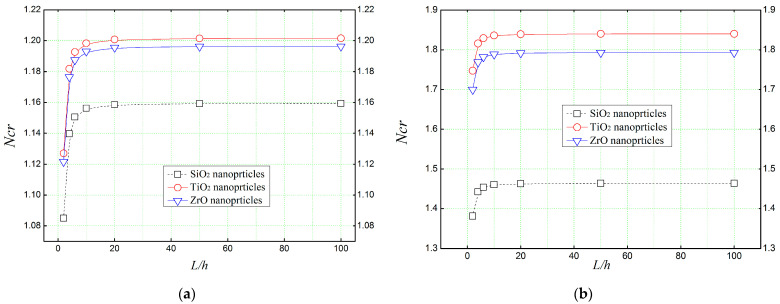
Thickness-to-length ratios effect on the critical buckling load of concrete beams reinforced with different amorphous nanoparticles. (**a**) *Vr* = 5%, (**b**) *Vr* = 30%.

Figure 8 shows the effect of thickness-to-length ratio (*L*/*h*) on the dimensionless critical buckling load (*N_cr_*) of a simply supported reinforced concrete beam (Figure 8a: *V_r_* = 5%, Figure 8b: *V_r_* = 30%) subjected to a uni-axial load (*N_0_*). The results related to Reddy’s refined third-order deformation theory (TSDT) are exhibited for different types of nano-sized reinforcements. It is quite clear from the results presented in Figure 8, that all the nanoparticulated beams have the same behavior when varying the geometrical ratios (*L*/*h*), in which, the critical buckling load starts to become stable when *L*/*h* is greater than 20. In addition, by establishing an analogy between Figure 8a,b, it can be noted that reinforcing the concrete beam with a proportion of 30% of nanoparticles can moderate the mechanical resistance of the beam.

**Figure 9:**
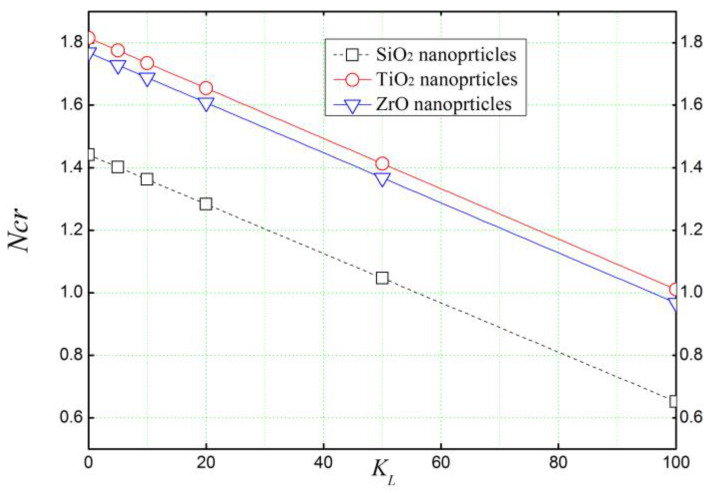
The effect of the lower spring stiffness *K_L_* (Winkler’s parameter) of the elastic foundation on the critical buckling load of reinforced concrete beams (*L/h =* 4, *K_U_ =* 0).

Figure 9 illustrates the effect of the parameter of Winkler springs *K_L_* on the critical buckling load of a concrete beam reinforced with different types of nanoparticles. The beam is assumed to be subjected to in-plane compressive loads ($N_{0}$). It can be seen that the spring constant *K_L_* has a significant effect on the critical buckling loads ($N_{cr}$) by reducing them depending on the type of reinforcement used in the concrete matrix.

**Figure 10:**
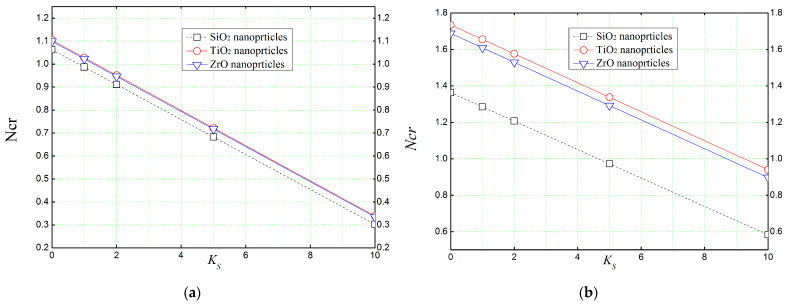
The effect of the shear layer *K_s_* (Pasternak’s parameter) of the elastic foundation on the critical buckling load of reinforced concrete beams (*L*/*h* = 4, *K_L_* = 10). (**a**) *Vr* = 5%, (**b**) *Vr* = 30%.

Figure 10 shows the effect of the Pasternak parameter *K_S_* of the elastic foundation on the critical buckling load of reinforced concrete beams. It is clear from Figure 10 that the shear layer parameter *K_S_* reduces the critical buckling loads required to make the beam unstable. Furthermore, as mentioned earlier, the use of 30% (Figure 10b) of nanoparticles in a concrete matrix makes the beam even more resistant to external mechanical buckling loads.

It can be seen from Table 5 that the shear layer (*K_S_*) and the upper spring parameters (*K_U_*) have a sliding effect on the non-dimensional critical buckling loads. Most especially, the shear layer parameter (*K_S_*) appears to be more effective than the upper spring constant. It can also be inferred that the presence of an elastic Kerr foundation significantly affects the buckling response of the reinforced concrete beam, thus resulting in a decrease in buckling loads with increasing stiffness components.

### 5.3. Dynamic Analysis

With the following, the free vibrational analysis of reinforced concrete beams is analytically studied. Natural frequencies ($\hat{\omega }$) of titanium dioxide (TiO_2_) and zirconium oxide (ZrO) enhanced concrete beam is disclosed in Table 6, the volume concentrations (*Vr*) of titanium oxide is confined between 0% (for a non-reinforced concrete beam) and 30%. Several beam theories are used to provide accurate results.

It can be observed from Table 6 that the different higher beam theories (TSDT, TrSDT, ESDT) used in the dynamic analysis are in fair accordance when estimating the non-dimensional natural frequencies ($\hat{\omega }$) of the reinforced concrete beam, while the first-order and the classical shear deformation theories give less accurate results. It is to be pointed that the maximum values of the dimensionless natural frequency are obtained when the volume concentrations of the reinforcements are around 30%. Moreover, in Table 6, several beam theories were employed to determine the dimensionless free vibration frequencies ($\hat{\omega }$) of a concrete beam strengthened with different proportions of TiO_2_ and ZrO nanoparticles. It can be seen that zirconium oxide volumes have a slight effect on increasing the natural frequencies of the nanocomposite concrete beam compared to the titanium ones.

**Figure 11:**
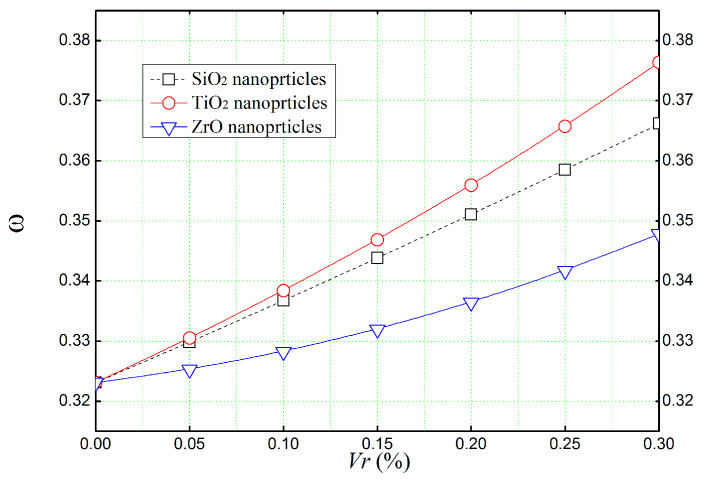
The non-dimensional natural frequencies ($\hat{\omega }$) of simply supported concrete beams reinforced with several types of nanoparticles (*L*/*h* = 4).

The effect of different volume fractions (*V_r_*) of silica dioxide (SiO_2_), titanium dioxide (TiO_2_), and zirconium oxide (ZrO) on natural frequencies ($\hat{\omega }$) of a simply supported concrete beam was displayed in Figure 11 using Reddy’s third-order shear deformation theory (TSDT). It can be apparent that the volume concentration of nanoparticles tends to increase the natural frequencies of the reinforced concrete beams. In particular, the zirconium oxide (ZrO) reinforced concrete beam has the lowest natural frequencies, mainly due to height material density of ZrO.

**Figure 12:**
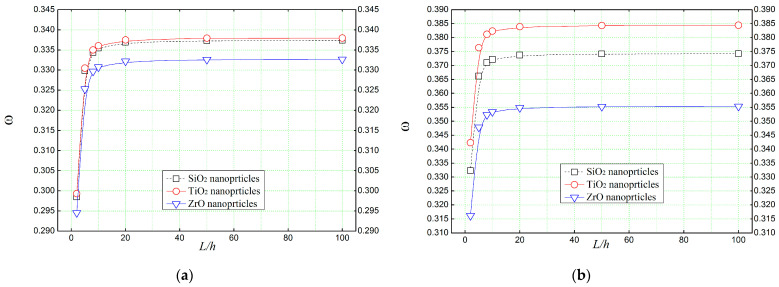
Thickness-to-length ratios effect on the natural frequency ($\hat{\omega }$) of concrete beams reinforced with different amorphous nanoparticles. (**a**) *Vr* = 5%, (**b**) *Vr* = 30%.

Figure 12 shows the effect of thickness-to-length ratio (*L*/*h*) on the non-dimensional frequency of concrete beams reinforced with various types of amorphous nanoparticles. As noted before, among those RC beams, the one enhanced with zirconium oxide nanoparticles has the lower natural frequencies compared to the ones enhanced with titanium or silica nanoparticles.

**Figure 13:**
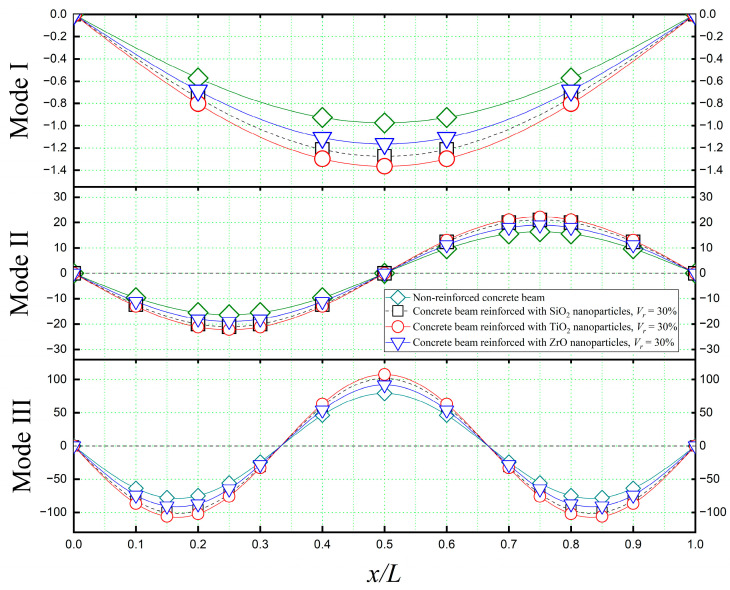
The first three mode shapes of a concrete beam reinforced with various types of nanoparticles (*L*/*h* = 10, *K_U_* = 0, *K_S_* = 0).

Figure 13 presents a comparison of mode shapes obtained from the free vibrational analysis of a simply supported concrete beam. The beam is reinforced with various nanoparticles, each comprising 30% of the total volume of the matrix. These mode shapes are contrasted with those of a non-reinforced concrete beam, highlighting the distinctive vibrational behavior introduced by the nanoparticles’ incorporation. The resulting mode shapes depicted in Figure 13 are referred to as doubly coupled modes according to Equation (5b), which involves substantial shear and flexure deformations.

**Figure 14:**
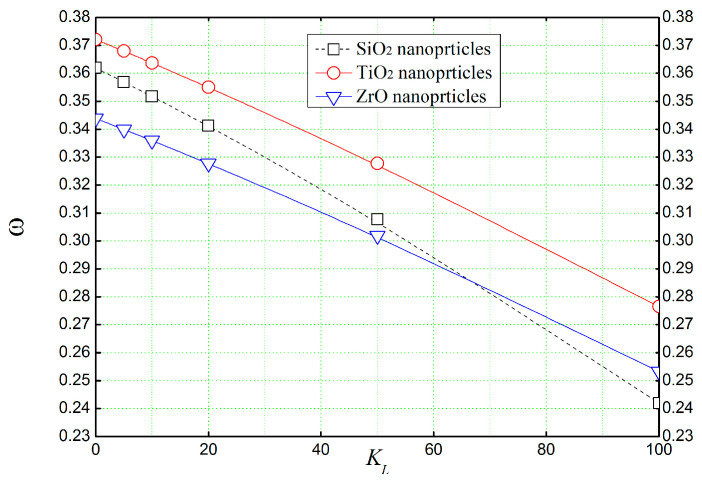
The effect of lower spring stiffness *K_L_* (Winkler’s parameter) of the elastic foundation on the natural frequency ($\hat{\omega }$) of reinforced concrete beams (*L*/*h* = 4, *K_U_* = 0, *K_S_* = 0).

Figure 14 shows the effect of Winkler’s parameter *K_L_* (spring constant) on the non-dimensional frequency ($\hat{\omega }$) of a concrete beam reinforced with different types of nano-sized particles. It can be observed that the spring constant *K_L_* has a sliding effect on the non-dimensional frequencies ($\hat{\omega }$), as the frequencies are decreased by increasing the value of the spring constant. More interestingly, when Winkler’s spring constant exceeds the value of 65, one can observe that the natural frequencies of a concrete beam reinforced with ZrO surpass those of a concrete beam reinforced with SiO_2_.

**Figure 15:**
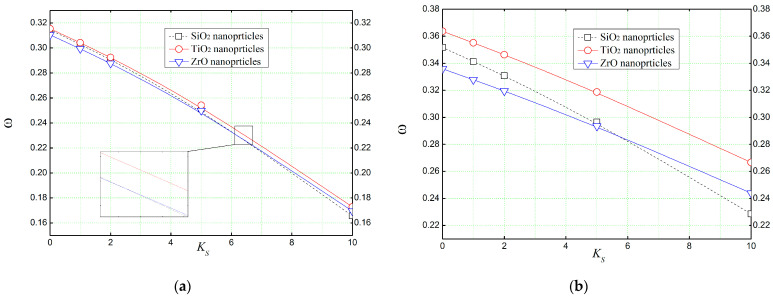
The effect of the shear layer *K_S_* (Pasternak’s parameter) of the elastic foundation on the natural frequency ($\hat{\omega }$) of reinforced concrete beams (*L*/*h* = 4, *K_L_* = 10, *K_U_* = 0). (**a**) *Vr* = 5%, (**b**) *Vr* = 30%.

With reference to Figure 15, the effect of Pasternak’s shear layer constant *K_S_* on the dimensionless frequencies ($\hat{\omega }$) of a concrete beam reinforced with various nano-scale particles is illustrated. It is quite clear that the shear layer constant also reduces the non-dimensional natural frequencies. This can be attributed to the effects of the elastic foundation on the load distribution along the beam.

Table 7 represents the effect of the shear layer and the upper spring constant on the dimensionless frequencies ($\hat{\omega }$) of RC beams, wherein it can be noticed that the shear layer (*K_S_*) and the upper spring parameters (*K_U_*) have a reducing effect on the frequencies. The shear layer parameter (*K_S_*) seems to have more effect than the upper spring constant. It can also be concluded that the presence of elastic Kerr foundation notably affects the dynamic behavior of reinforced concrete beams. By redistributing the forces of vibration, the foundation reduces the frequencies acting on the beam, improving its structural behavior.

## 6. Conclusions

The pursuit of improving construction concrete has driven the incorporation of nanomaterials, resulting in nano-reinforced concretes with enhanced mechanical performance and increased density. This response addresses the demand for advanced materials in the construction industry, promoting sustainable and resilient infrastructure development.

In our study, we focused on investigating the buckling and dynamic analyses of concrete beams reinforced with various types of nanoparticles. Employing Eshelby’s model, we accurately characterized the properties of the composite material. Utilizing a higher-order shear deformation theory, analytical results were obtained for the beam simulations.

Our investigation yielded significant insights and led to noteworthy conclusions:

1. The improvement offered by nanoparticles in concrete is primarily influenced by their mechanical characteristics. Higher mechanical characteristics of nanoparticles result in greater improvements in the nanocomposite.
2. Various parameters can affect the mechanical performance of nano-concrete, including the volume percentage of nanoparticles. The concentration of nanoparticles plays a crucial role in determining the mechanical properties of the composite material.
3. Geometrical aspect ratios have a significant impact on the buckling and vibration behavior of nano-reinforced concretes. The critical buckling loads and eigenvalues of the structure are influenced by the proportions and dimensions of the components in the composite system.
4. Higher-order deformation theories consistently provide consistent findings when analyzing nano-reinforced concretes. These theories are effective in capturing the complex behavior and accurately predicting the mechanical response of the composite material.
5. The presence of an elastic foundation has a substantial influence on the buckling load and frequency of reinforced concrete beams. The characteristics of the foundation, such as stiffness and boundary conditions, significantly affect the structural behavior of the system.
6. Our investigation revealed that titanium dioxide significantly improves mechanical properties against mechanical loads, but may reduce natural frequency and dynamic resilience. In contrast, zircon appears to offer the best overall performance as reinforcement against both mechanical and dynamic loads.

However, it is essential to acknowledge the limitations of our research, which focused solely on nano-titanium, nano-silica, and nano-zirconium. To broaden our understanding and encompass a wider range of structural elements, future investigations should explore the effects of alternative nano-reinforcements on the properties of concrete beams, plates, walls, and other structural components. Moreover, an exploration of alternative boundary conditions will be imperative. This will provide a more comprehensive understanding of their potential and pave the way for further advancements in the field of reinforced concrete structures.

## Figures and Tables

**Table 1:** Several higher-order shear deformation theories.

Theories	Shape Function
Third-order shear deformation theory (TSDT) [33]	$f\left(z\right)=z\left(1-\frac{4z^{2}}{3h^{2}}\right)$
Trigonometric shear deformation theory (TrSDT) [34]	$f(z)=\frac{h}{\pi }\sin\left(\frac{\pi z}{h}\right)$
Exponential shear deformation theory (ESDT) [35]	$f\left(z\right)=ze^{-2{\left(z/h\right)}^{2}}$
First-order shear deformation theory [36]	$f\left(z\right)=z$
Classical beam theory [37]	$f\left(z\right)=0$

**Table 2:** Physical characteristics of the nanoparticles used in the study.

Cementitious Materials	SiO_2_	TiO_2_	ZrO
*E_r_*: Young’s elastic moduli (GPa)	70	282	210
ρ: Density (Kg/m^3^)	2650	4230	5610
*υ*: Poisson’s ratio	0.17	0.28	0.31

**Table 3:** Validation of the present theory against other published work.

Theory	Shape Function *f*(*z*)	Lh=20		
		$\bar{w}$	$\bar{\sigma_{x}}$	$\bar{\tau_{xz}}$
Present	$f\left(z\right)=z\left(1-\frac{4z^{2}}{3h^{2}}\right)$	8.2591	12.1669	0.4773
Harrat et al. [10]	$f\left(z\right)=z\left[\frac{-1}{4}+\frac{5}{3}{\left(\frac{z}{h}\right)}^{2}\right]$	8.2591	12.1669	0.4774
TrSDT [34]	$f(z)=\frac{h}{\pi }\sin\left(\frac{\pi z}{h}\right)$	8.2591	12.1668	0.4760
Timoshenko (FSDT) [36]	$f\left(z\right)=z$	8.2251	12.2087	0.3819
Euler–Bernoulli (CBT) [37]	$f\left(z\right)=0$	8.2534	12.1585	0.3183

**Table 4:** Dimensionless critical buckling loads of concrete beam reinforced with different proportions of nanoparticles (*L*/*h* = 10).

Beam Theory	Shape Function	$N_{cr}$						
		Non-Reinforced	TiO_2_ Volume *V_r_*			ZrO Volume *V_r_*		
		0%	5%	15%	30%	5%	15%	30%
TSDT [33]	$f\left(z\right)=z\left(1-\frac{4z^{2}}{3h^{2}}\right)$	1.1040	1.1984	1.4153	1.8361	1.1931	1.3971	1.7889
TrSDT [34]	$f(z)=\frac{h}{\pi }\sin\left(\frac{\pi z}{h}\right)$	1.1036	1.1980	1.4149	1.8356	1.1927	1.3966	1.7884
ESDT [35]	$f\left(z\right)=ze^{-2{\left(z/h\right)}^{2}}$	1.1032	1.1976	1.4145	1.8351	1.1923	1.3962	1.7879
FSDT [36]	$f\left(z\right)=z$	1.0884	1.1826	1.3986	1.8166	1.1773	1.3802	1.7693
CBT [37]	$f\left(z\right)=0$	1.1072	1.2016	1.4187	1.8400	1.1964	1.4005	1.7928

**Table 5:** Dimensionless critical buckling loads of reinforced concrete beam resting on Kerr’s elastic foundation (*K_L_* = 10).

Lh	(*K_U_*_,_ *K_S_*)	$N_{cr}$								
		SiO_2_ Volume *Vr*			TiO_2_ Volume *Vr*			ZrO Volume *Vr*		
		5%	15%	30%	5%	15%	30%	5%	15%	30%
4	(10, 0)	1.1012	1.2124	1.4023	1.1431	1.3582	1.7755	1.1378	1.3400	1.7283
10		1.1111	1.2233	1.4147	1.1532	1.3700	1.7907	1.1480	1.3518	1.7435
20		1.1125	1.2248	1.4165	1.1547	1.3717	1.7929	1.1495	1.3535	1.7457
100		1.1130	1.2253	1.4171	1.1552	1.3723	1.7936	1.1499	1.3541	1.7464
4	(10, 5)	0.9112	1.0201	1.2073	0.9518	1.1631	1.5767	0.9468	1.1455	1.5306
10		0.8885	1.0003	1.1913	0.9304	1.1466	1.5666	0.9252	1.1285	1.5196
20		0.8852	0.9974	1.1890	0.9273	1.1442	1.5652	0.9221	1.1260	1.5180
100		0.8841	0.9965	1.1882	0.9263	1.1434	1.5647	0.9211	1.1252	1.5175
4	(10, 10)	0.7212	0.8278	1.0124	0.7605	0.9679	1.3780	0.7558	0.9509	1.3328
10		0.6659	0.7773	0.9679	0.7076	0.9231	1.3426	0.7024	0.9051	1.2957
20		0.6578	0.7699	0.9614	0.6999	0.9166	1.3374	0.6947	0.8984	1.2903
100		0.6552	0.7676	0.9593	0.6974	0.9145	1.3358	0.6922	0.8963	1.2886

**Table 6:** Dimensionless natural frequencies (ω) of concrete beam reinforced with different proportions of nanoparticles (*L*/*h* = 10).

**Beam Theory**	Shape Function	(ω)						
		Non-Reinforced	TiO_2_ Volume *V_r_*			ZrO Volume *V_r_*		
		0%	5%	15%	30%	5%	15%	30%
TSDT [33]	$f\left(z\right)=z\left(1-\frac{4z^{2}}{3h^{2}}\right)$	0.3287	0.3362	0.3526	0.3825	0.3309	0.3375	0.3535
TrSDT [34]	$f(z)=\frac{h}{\pi }\sin\left(\frac{\pi z}{h}\right)$	0.3287	0.3361	0.3526	0.3824	0.3308	0.3375	0.3535
ESDT [35]	$f\left(z\right)=ze^{-2{\left(z/h\right)}^{2}}$	0.3286	0.3361	0.3525	0.3824	0.3308	0.3374	0.3534
FSDT [36]	$f\left(z\right)=z$	0.3265	0.3340	0.3420	0.3805	0.3287	0.3355	0.3516
CBT [37]	$f\left(z\right)=0$	0.3292	0.3366	0.3445	0.3829	0.3313	0.3379	0.3539

**Table 7:** Dimensionless frequencies (ω) of reinforced concrete beam resting on Kerr’s elastic foundation (*K_L_* = 10).

Lh	(*K_U_*, *K_S_*)	(ω)								
		SiO_2_ Volume *Vr*			TiO_2_ Volume *Vr*			ZrO Volume *Vr*		
		5%	15%	30%	5%	15%	30%	5%	15%	30%
4	(10, 0)	0.3209	0.3350	0.3575	0.3217	0.3494	0.3703	0.3166	0.3239	0.3405
10		0.3289	0.3434	0.3665	0.3298	0.3469	0.3777	0.3246	0.3320	0.3490
20		0.3302	0.3446	0.3678	0.3310	0.3482	0.3791	0.3258	0.3332	0.3503
100		0.3306	0.3451	0.3683	0.3314	0.3486	0.3795	0.3261	0.3336	0.3507
4	(10, 5)	0.2917	0.3070	0.3315	0.2933	0.3130	0.3471	0.2886	0.2993	0.3203
10		0.2941	0.3105	0.3363	0.2962	0.3174	0.3533	0.2914	0.3033	0.3258
20		0.2945	0.3110	0.3370	0.2966	0.3180	0.3542	0.2918	0.3039	0.3267
100		0.2946	0.3112	0.3372	0.2967	0.3182	0.3545	0.2919	0.3041	0.3269
4	(10, 10)	0.2593	0.2764	0.3034	0.2620	0.2854	0.3244	0.2576	0.2725	0.2987
10		0.2546	0.2737	0.3031	0.2583	0.2848	0.3270	0.2539	0.2717	0.3009
20		0.2539	0.2733	0.3030	0.2577	0.2846	0.3274	0.2532	0.2715	0.3012
100		0.2536	0.2731	0.3030	0.3217	0.3494	0.3703	0.3166	0.3239	0.3405

## Data Availability

The data presented in this study are available on request from the corresponding author.
